# A study on the correlations of PRL levels with anxiety, depression, sleep, and self-efficacy in patients with prolactinoma

**DOI:** 10.3389/fendo.2024.1369729

**Published:** 2024-03-19

**Authors:** Xiaoju Miao, Zhongmin Fu, Xian Luo, Jun Wang, Lili Yuan, Shunjun Zhao, Yi Feng, Shiming Huang, Shunwu Xiao

**Affiliations:** ^1^ Department of Nursing, Affiliated Hospital of Zunyi Medical University, Zunyi, China; ^2^ The First Ward of the Neurosurgery Department, Affiliated Hospital of Zunyi Medical University, Zunyi, China

**Keywords:** prolactinoma, PRL levels, generalized estimating equations, anxiety, depression, sleep disorders, self-efficacy

## Abstract

**Purpose:**

The purpose of this study was to explore the factors influencing PRL levels in patients with prolactinoma and to investigate the correlations between anxiety, depression, sleep, self-efficacy, and PRL levels.

**Methods:**

This retrospective study included 176 patients with prolactinoma who received outpatient treatment at the Affiliated Hospital of Zunyi Medical University from May 2017 to August 2022. The general information questionnaire, Hospital Anxiety and Depression Scale (HADS), Athens Insomnia Scale (AIS), and General Self-Efficacy Scale (GSES) were used for data collection. A generalized estimating equation (GEE) model was used to analyze the factors influencing PRL levels in patients with prolactinoma. GEE single-effect analysis was used to compare PRL levels at different time points between anxiety group and nonanxiety group, between insomnia group and normal group, and between low, medium, and high self-efficacy groups.

**Results:**

The median baseline PRL level and the PRL levels at 1, 3, 6, and 12 months of follow-up were 268.50 ng/ml, 122.25 ng/ml, 21.20 ng/ml, 19.65 ng/ml, and 16.10 ng/ml, respectively. Among patients with prolactinoma, 59.10% had anxiety (HADS-A score = 7.35 ± 3.34) and 28.98% had depression (HADS-D score = 5.23 ± 3.87), 9.10% had sleep disorders (AIS score = 6.10 ± 4.31) and 54.55% had low self-efficacy (GSES score = 2.13 ± 0.83). Educational level, tumor size, number of visits, sleep quality, anxiety level, and self-efficacy level were found to be factors influencing PRL levels in patients with prolactinoma (P<0.05). Higher PRL levels were observed in the anxiety group compared to the non-anxiety group (P<0.001), in the insomnia group compared to the normal group (P<0.05), and in the low self-efficacy group compared to the medium and high self-efficacy groups (P<0.05).

**Conclusion:**

PRL levels in patients with prolactinoma are related to education level, tumor size, number of visits, anxiety, self-efficacy, and sleep but not depression. PRL levels were higher in patients with anxiety, low self-efficacy, and sleep disorders.

## 1 Introduction

Prolactinoma is a common pituitary adenoma that occurs in individuals with hypothalamic-pituitary disease and is characterized by excessive secretion of prolactin (PRL) from anterior pituitary lactotroph cells. The incidence rate of prolactinoma is approximately 50 per 100,000 individuals, with an annual new case rate of 3 to 5 per 100,000 (1). The typical clinical manifestations of prolactinoma include menstrual disorders, galactorrhea, infertility, decreased libido, erectile dysfunction, hypogonadism and pituitary mass lesions (headache, partial hypopituitarism, visual field disturbances), all of which significantly impact the quality of life and mental health of affected patients (2). Currently, the clinical management of pituitary adenomas primarily consists of pharmacotherapy, surgery, and radiation therapy. The first-line pharmacological treatment for prolactinoma includes dopamine agonists (DAs), such as bromocriptine or cabergoline (3), which aim to suppress excessive PRL secretion and reduce tumor size using the minimum effective dose.

However, the relationships between PRL and anxiety and depression in prolactinoma patients remain to be explored. It has been reported that prolactinoma patients may experience dissatisfaction with their body image due to weight gain, and symptomatic patients have a higher rate of body dissatisfaction than asymptomatic patients (4). Prolactinoma patients often exhibit symptoms of depression, hostility, irritability, and anxiety (5, 6), and stress may trigger neuroendocrine changes involving dopamine or serotonin, thus affecting the release of PRL (7). The study demonstrated that compared to low anxiety behavior (LAB) rats, high anxiety behavior (HAB) rats exhibited increased basal and stress-induced PRL levels, possibly due to the association between inborn anxiety and HPA axis hyper-reactivity (8). High levels of PRL may hinder the formation of the homodimers required for PRL’s physiological functions. Persistent hyperprolactinemia reduces the ability of tuberoinfundibular neurons to make dopamine (9). Increased PRL levels result in higher levels of vasoinhibins, which could contribute to anxiety and depression behaviors (10, 11). However, studies by Sonino et al. have shown that even after restoring PRL levels to normal through treatment, patients may still experience persistent psychological issues, suggesting that the optimal endocrine balance may not be fully restored (12). However, the relationship between PRL levels in prolactinoma patients and sleep disturbances remains unclear. Some studies have indicated that there is no direct relationship between sleep disturbances and PRL levels in prolactinoma patients (13), but a retrospective study has reported a possible association between elevated PRL levels and excessive daytime sleepiness (14). In terms of self-efficacy, multiple studies have shown that patients with high levels of self-efficacy are more confident in coping with adversity or illness and are more likely to adopt a positive and healthy attitude toward them (15, 16). However, there is currently no research available on the relationship between PRL levels and self-efficacy in prolactinoma patients.

In summary, the correlations of PRL levels with anxiety, depression, sleep disturbances, and self-efficacy in prolactinoma patients have not been thoroughly investigated. Therefore, this study used a generalized estimating equation (GEE) to analyze the one-year follow-up data of prolactinoma patients and explore the factors influencing their PRL levels. Additionally, this study investigated the correlations between anxiety, depression, sleep disturbances, self-efficacy, and PRL levels in prolactinoma patients, thus providing evidence for targeted interventions to improve the quality of life and psychological status of prolactinoma patients.

## 2 Methods

### 2.1 Study subjects

This study included patients with prolactinoma who received outpatient treatment at the Affiliated Hospital of Zunyi Medical University from May 2017 to August 2022. The patients were divided into three groups: 128 patients with microadenomas, 38 patients with macroadenomas, and 10 patients with giant adenomas.

### 2.2 Inclusion and exclusion criteria

The inclusion criteria were as follows: 1) aged older than 18 years, 2) had complete clinical data, and 3) had a confirmed diagnosis of prolactinoma. The exclusion criteria were as follows: 1) had complications in vital organs such as the heart, brain, or kidneys; 2) were unable to cooperate with assessments or had a history of cognitive impairment or psychiatric disorders; 3) were pregnant; and 4) had significant missing data on the core variables of interest in this study.

### 2.3 Research tools

A general information questionnaire was used, which included demographic information (sex, age at initial diagnosis of prolactinoma, education level, living arrangement, reproductive status, monthly family income, BMI, and number of hospital admissions) and disease-related information (recurrence of prolactinoma, tumor size, chronic diseases, occurrence of hyperprolactinemia during follow-up, Knosp grade, pretreatment prolactin levels, and prolactin levels at 1 month, 3 months, 6 months, and 1 year of follow-up). Prolactin levels at 1 month, 3 months, 6 months, and 1 year of follow-up were measured during routine examinations when patients visited the outpatient clinic. All data and information were collected retrospectively through the medical record system.

The Hospital Anxiety and Depression Scale (HADS) is a scale developed by Zigmond and Snaith in 1983 to assess anxiety and depression in pregnant patients in clinical or hospital settings (17, 18). The HADS consists of two parts: the Anxiety subscale (HADS-A) and the Depression subscale (HADS-D), each with 7 items. Each item is scored on a 4-point Likert scale, and the total score for each subscale ranges from 0 to 21. A cutoff score of 7 was used to determine the presence of anxiety or depression, with scores between 8 and 10 indicating mild anxiety or depression, scores between 11 and 14 indicating moderate anxiety or depression, and scores between 15 and 21 indicating severe anxiety or depression.

The Athens Insomnia Scale (AIS) was used to measure sleep quality in patients during the study period (19). The AIS is an internationally recognized self-report measure of sleep quality that consists of 8 items. Each item is scored on a 4-point Likert scale ranging from 0 to 3, with 0 indicating “no problem” and 3 indicating “severe problem”. The total score ranges from 0 to 24, with scores above 4 indicating the presence of sleep disorders. Higher scores indicate lower sleep quality and more severe sleep problems.

The General Self-Efficacy Scale (GSES), developed by Schwarzer et al., is widely used internationally (20). The Chinese version of the GSES has also demonstrated good applicability (21). We assessed the self-efficacy levels of prolactinoma patients using the Chinese version of the GSES. The Chinese version of the GSES consists of 10 items scored on a 4-point Likert scale. A higher total score indicates a stronger sense of self-efficacy. Based on the average self-efficacy scores, individuals can be classified into low-level self-efficacy (1.0-2.0), moderate-level self-efficacy (2.1-3.0), and high-level self-efficacy (3.1-4.0).

The HADS, AIS, and GSES were all collected retrospectively by the researchers from the outpatient medical record system.

### 2.4 Terminology definitions

BMI was calculated as body weight (kg)/height^2^ (m^2^). A BMI ≤18.5 indicates underweight, 18.6-23.9 indicates normal weight, ≥24 indicates overweight, and ≥28 indicates obesity (22). Pituitary prolactinomas are classified based on tumor size as microadenomas (≤1 cm), macroadenomas (1-4 cm), or giant adenomas (>4 cm)^3^. Knosp et al. (23) classified pituitary adenomas (PAs) into four grades based on the relative position of the tumor lateral to the internal carotid artery on MRI, using the mid-sagittal plane of the sella turcica as a reference and marking the inner, middle, and outer lines between the intracavernous segment of the internal carotid artery and the flow void. According to the Knosp classification, Grade 3 PAs exhibit tumor borders extending beyond the outer line of the internal carotid artery, while Grade 4 PAs encase the internal carotid artery, thus defining invasive PAs.

The detection method for PRL was as follows. Fasting blood samples were collected from patients during outpatient visits to monitor of PRL levels. PRL detection was performed using an Elecsys Prolactin II assay kit. The reference ranges were as follows: female, 4.79-23.30 ng/mL; male, 4.04-15.20 ng/mL. Patients with serum PRL levels above 23.30 ng/mL were diagnosed with hyperprolactinemia (according to the reference values provided by the PRL assay kit used in this study).

### 2.5 Statistical analysis

Normally distributed continuous variables are described using means and standard deviations (SDs), while nonnormally distributed continuous variables are described using medians (Ms) and interquartile ranges (IQRs). Categorical variables are described using frequencies and percentages. Since PRL exhibits a nonnormal distribution and is a repeated measurement variable, generalized estimating equation (GEE) models were used to analyze the factors influencing PRL levels in patients with pituitary prolactinoma. PRL was included as a continuous variable in the GEE model, with the Gaussian distribution selected for its distribution and the identity link function selected for its connection function. The independent variables were included in the GEE model as dummy variables to identify the factors affecting PRL levels. Furthermore, we compared the levels of PRL at different time points in the anxiety group versus the nonanxiety group, the sleep disorder group versus the nonsleep disorder group, and the low, medium, and high self-efficacy groups using separate effect analysis in the Generalized Estimating Equation (GEE) model. All the statistical analyses were performed using Stata 17 software, and the graphs were generated using GraphPad Prism 9.0. The significance level was set at two-sided (α=0.05), with *P* < 0.05 indicating statistical significance.

## 3 Results

### 3.1 Basic characteristics of patients

A total of 176 patients with prolactinoma, 30 males and 146 females, participated in this study. 2.2% of patients was over 60 years old. 29.5% of patients had a Bachelor and above education degree. 52.3% of the patients live in rural areas for a long time. 52.8% of the patients had reproductive capacity. 52.3% of the patients had a monthly family income of less than 5000 yuan. 77.3% of the patients had a BMI≥24. 61.9% of the patients had subsequent visits. 56.8% of patients experienced prolactinoma recurrence. The majority of patients (72.7%) had microadenomas, and 58.5% of patients had chronic diseases. A Knosp grade 3 or above was present in 30.7% of patients, and 4 patients reported hyperprolactinemia during the treatment process. The mean HADS-A score for prolactinoma patients was 7.35 ± 3.34, with 59.10% of patients experiencing anxiety. The mean HADS-D score was 5.23 ± 3.87, and 28.98% of patients had depression. The AIS score was 6.10 ± 4.31, with 59.10% of patients having sleep disorders. The GSES score was 2.13 ± 0.83, and 54.55% of patients had low self-efficacy levels. The PRL levels showed a marked decrease in the first month of treatment, followed by stabilization of the concentration. The detailed results are presented in **Table 1**.

**Table 1:** Baseline characteristics.

Variable	value
Female, n(%)	146 (83)
Age, n(%)	
18-39y	81 (46)
40-60y	56 (31.8)
>60y	39 (22.2)
Education, n(%)	
Primary school and below	26 (14.8)
Middle school	26 (14.8)
High school	36 (20.5)
Junior college	36 (20.5)
Bachelor and above education degree	52 (29.5)
Residential types, n(%)	
Urban	84 (47.7)
Rural	92 (52.3)
Fertility outcome, n(%)	
Fertility	93 (52.8)
Infertility	83 (47.2)
Monthly family income, n(%)	
<5 000yuan	92 (52.3)
5 000~10 000yuan	57 (32.4)
>10 000yuan	27 (15.3)
BMI(kg/m2), n(%)	
<18.5	7 (4.0)
18.5-23.9	33 (18.8)
24-27.9	97 (55.1)
≥28	39 (22.2)
Hospital visits, n(%)	
first visit	67 (38.1)
subsequent visit	109 (61.9)
Recurrence of prolactinoma	
recurrence	100 (56.8)
no recurrence	76 (43.2)
Tumor size, n(%)	
microadenoma	128 (72.7)
macroadenoma	38 (21.6)
giant adenoma	10 (5.7)
chronic diseases, n(%)	
existence	103 (58.5)
nonexistence	73 (41.5)
Hyperprolactinemia during the treatment process, n(%)	
existence	4 (2.3)
nonexistence	172 (97.7)
Knosp grade, n(%)	
0	25 (14.2)
I	34 (19.3)
II	63 (35.8)
III	45 (25.6)
IV	9 (5.1)
HADS-A,mean(SD)	7.35 (3.34)
HADS-D, mean (SD)	5.23 (3.87)
The Athens Insomnia Scale(AIS), mean (SD)	6.10 (4.31)
General Self-Efficacy Scale(GSES), mean (SD)	2.13 (0.83)
PRL(ng/ml), M(IQR)	
baseline	268.50 (124.00)
1 month	122.25 (9.80)
3 months	21.20 (7.22)
6 months	19.65 (8.20)
1 year	16.10 (7.88)

### 3.2 Factors influencing PRL levels in patients with prolactinoma

The GEE model showed that education level, tumor size, number of visits, sleep quality, anxiety level, and self-efficacy level were significantly related to PRL levels (*P* < 0.05, **Table 2**). Compared to patients with a Bachelor’s degree or higher education level, patients with a primary school or below (*P* = 0.038), middle school (*P* = 0.013), and high school education level (*P* = 0.003) had higher levels of PRL. Compared to patients with giant adenoma, patients with microadenoma (*P* = 0.017), macroadenoma (*P* = 0.036) had lower levels of PRL. Compared to patients who were visiting for the first time, those who had multiple visits for prolactinoma tended to have higher PRL levels (*P* = 0.029). Compared to patients with severe anxiety, those without anxiety (*P* < 0.001) and those with mild anxiety (*P* < 0.001) had lower PRL levels. The difference in PRL levels between patients with moderate anxiety (*P* = 0.054) and patients with severe anxiety was not statistically significant. Patients without sleep disorders had lower PRL levels than those with sleep disorders (*P* = 0.003). Patients with low self-efficacy (*P* < 0.001) and moderate self-efficacy (*P* < 0.001) had higher PRL levels than patients with high self-efficacy.

**Table 2:** Analysis results of factors influencing PRL levels (ng/ml) in patients with prolactinoma.

Parameter	β	SE	95% CI		Waldχ^2^	*P*
			lower limit	upper limit		
intercept	132.061	7.949	116.481	147.642	275.990	<0.001
Time						
baseline	289.415	12.028	265.842	312.990	579.012	**<0.001**
1 month	98.225	1.609	95.072	101.379	3727.550	**<0.001**
3 months	5.675	0.313	5.062	6.288	329.184	**<0.001**
6 months	3.094	0.275	2.556	3.633	126.842	**<0.001**
1 year	0^a^	–	–	–	–	–
Gender						
male	0.273	1.975	-3.598	4.143	0.019	0.890
female	0^a^	–	–	–	–	–
Age						
18-39y	3.141	1.984	-0.747	7.030	2.507	0.113
40-60y	-0.459	2.173	-4.717	3.799	0.045	0.833
>60y	0^a^	–	–	–	–	–
Education						
Primary school and below	6.592	3.177	0.365	12.818	4.305	**0.038**
Middle school	7.009	2.813	1.495	12.523	6.207	**0.013**
High school	8.199	2.784	2.742	13.656	8.671	**0.003**
Junior college	-0.828	2.254	-5.246	3.591	0.135	0.714
Bachelor and above education degree	0^a^	–	–	–	–	–
Residential types						
Urban	2.003	1.480	-0.898	4.904	1.831	0.176
Rural	0^a^	–	–	–	–	–
Fertility outcome						
Fertility	-0.046	2.004	-3.975	3.883	0.001	0.982
Infertility	0^a^	–	–	–	–	–
Monthly family income						
<5 000yuan	1.817	2.7236	-3.521	7.155	0.445	0.505
5 000~10 000yuan	-0.246	2.4893	-5.125	4.633	0.010	0.921
>10 000yuan	0^a^	–	–	–	–	–
BMI(kg/m^2^)						
<18.5	-8.636	4.987	-18.410	1.138	2.999	0.083
18.5-23.9	0.366	2.411	-4.360	5.093	0.023	0.879
24-27.9	0.058	2.275	-4.402	4.517	0.001	0.980
≥28	0^a^	–	–	–	–	–
Hospital visits						
first visit	-3.764	1.720	-7.135	-0.394	4.791	**0.029**
subsequent visit	0^a^	–	–	–	–	–
Recurrence of prolactinoma						
recurrence	-1.971	1.638	-5.181	1.238	1.449	0.229
no recurrence	0^a^	–	–	–	–	–
Tumor size, n(%)						
microadenoma	-44.892	18.852	-81.842	-7.942	5.670	**0.017**
macroadenoma	-39.477	18.830	-76.383	-2.570	4.395	**0.036**
giant adenoma	0^a^	–	–	–	–	–
underlaying disease, n(%)						
existence	-2.584	1.550	-5.623	0.455	2.778	0.096
Nonexistence	0^a^	–	–	–	–	–
Hyperprolactinemia during the treatment process, n(%)						
existence	1.653	14.696	-27.151	30.457	0.013	0.910
Nonexistence	0^a^	–	–	–	–	–
Knosp grade, n(%)						
1	0.457	2.757	-4.947	5.860	0.027	0.868
2	-1.079	2.481	-5.941	3.783	0.189	0.664
3	0.207	2.805	-5.290	5.704	0.005	0.941
4	6.464	4.122	-1.614	14.542	2.459	0.117
0	0^a^	–	–	–	–	–
Anxiety status						
normal	-85.326	23.936	-132.239	-38.413	12.708	**<0.001**
mild anxiety	-82.513	23.615	-128.798	-36.229	12.209	**<0.001**
moderate anxiety	-24.400	12.667	-49.226	0.427	3.710	0.054
severe anxiety	0^a^	–	–	–	–	–
Depression status						
normal	-22.996	14.622	-51.654	5.662	2.473	0.116
mild depression	-28.735	15.732	-59.570	2.101	3.336	0.068
moderate depression	-17.466	14.563	-46.008	11.076	1.439	0.230
severe depression	0^a^	–	–	–	–	–
sleep quality						
normal	-9.827	3.320	-16.334	-3.320	8.762	**0.003**
Insomnia	0^a^	–	–	–	–	–
self-efficacy level						
low level	35.885	3.116	29.778	41.992	132.628	**<0.001**
medium level	30.108	2.459	25.289	34.926	149.968	**<0.001**
high level	0^a^	–	–	–	–	–

^a^This parameter was set to zero because it was redundant.

The bold values represent factors with statistical significance ( P < 0.05 ).

The symbol "-" indicates that the parameter serves as a reference object.

### 3.3 Comparison of PRL levels between the anxiety group and the nonanxiety group

**Figure 1:**
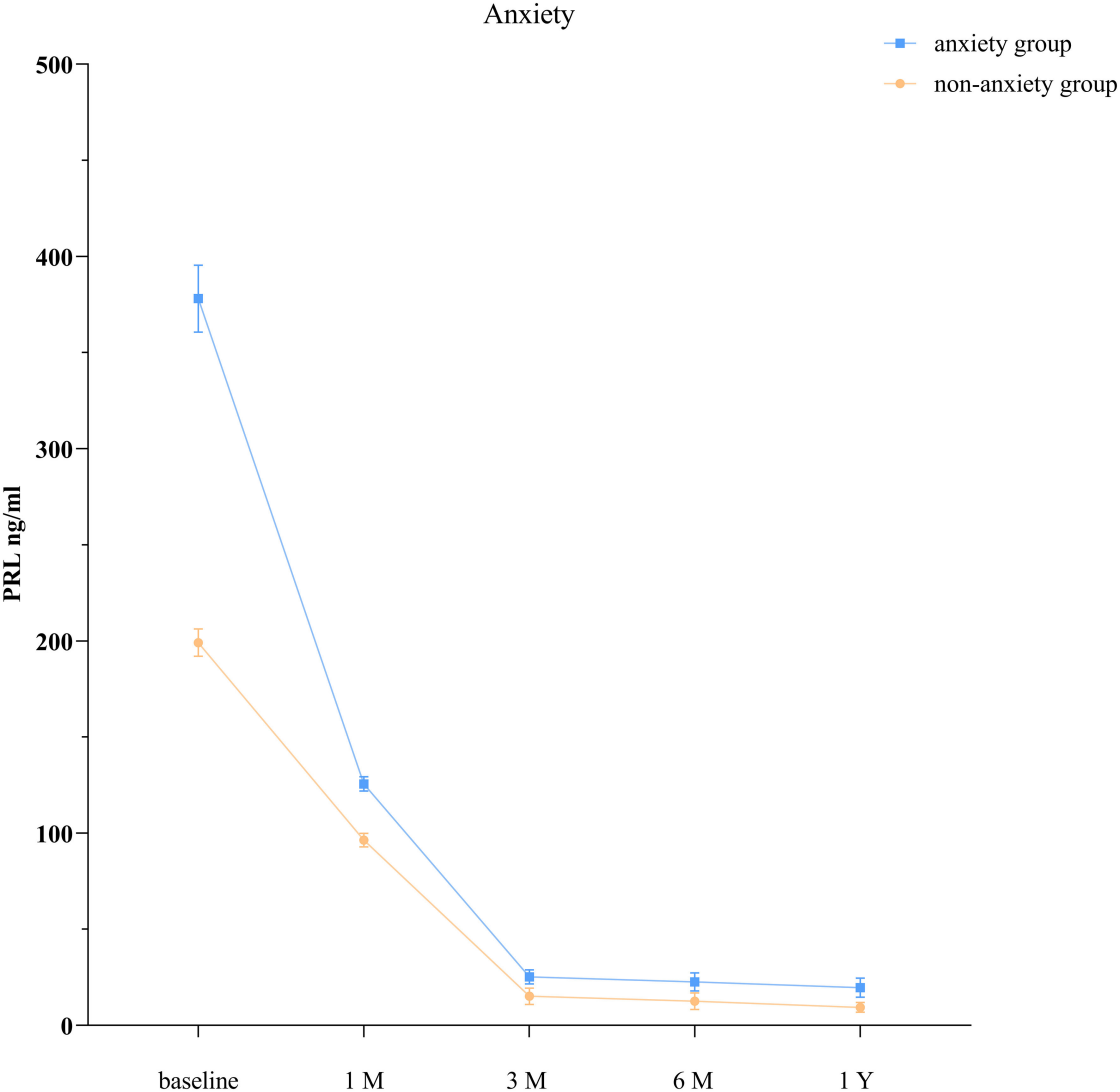
PRL levels in patients with prolactinoma with and without anxiety.

The GEE model showed that the anxiety group and the nonanxiety group had significantly different PRL levels at each time point (*P* < 0.001; **Table 3**, **Figure 1**).

**Table 3:** Comparison of PRL between the anxiety and nonanxiety groups.

Time	nonanxiety group(n=72) mean (SD)	anxiety group(n=104) mean (SD)	Between group differences (95% CI)	*P*
baseline	199.08 (7.10)	378.03 (17.42)	178.95(142.07,215.82)	<0.001
1 month	96.34 (3.51)	125.61 (3.73)	29.26(22.24, 36.28)	<0.001
3 months	15.13 (4.22)	25.21 (3.65)	10.07 (8.72,11.41)	<0.001
6 months	12.62 (4.32)	22.58 (4.67)	9.96 (8.56,11.35)	<0.001
1 year	9.36 (2.52)	19.59 (4.98)	10.23 (8.45,12.02)	<0.001

### 3.4 Comparison of PRL levels between the normal group and sleep disorders group

**Figure 2:**
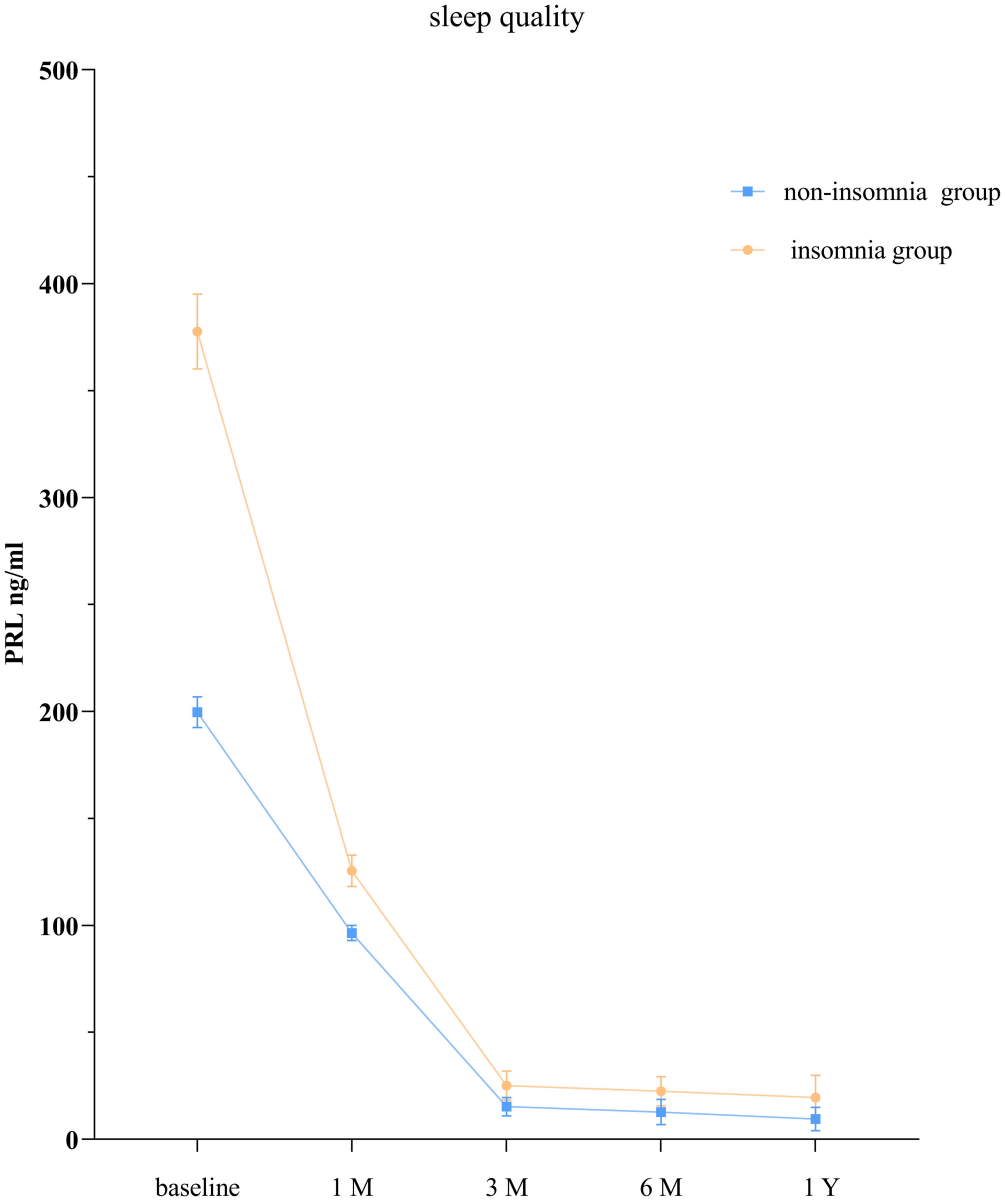
PRL levels in patients with prolactinoma with and without insomnia

The GEE model showed that the group without sleep disorders and the group with sleep disorders had significantly different PRL levels at each time point (*P* < 0.05; **Table 4**, **Figure 2**).

**Table 4:** Comparison of PRL between the normal and insomnia groups.

Time	normal group(n=72) mean (SD)	insomnia group(n=104) mean (SD)	Between group differences (95% CI)	*P*
baseline	199.64(7.19)	377.64(17.44)	178.01(141.02,214.99)	<0.001
1 month	96.42(3.51)	125.55(7.31)	29.13(22.10, 36.16)	0.001
3 months	15.24(4.26)	25.13(6.75)	9.89(8.52,11.25)	<0.001
6 months	12.72(5.89)	22.50(6.82)	9.78(8.36,11.21)	<0.001
1 year	9.47(5.44)	19.52(10.41)	10.05(8.24,11.86)	<0.001

### 3.5 Comparison of PRL levels among low self-efficacy group, moderate self-efficacy group, and the high self-efficacy group

**Figure 3:**
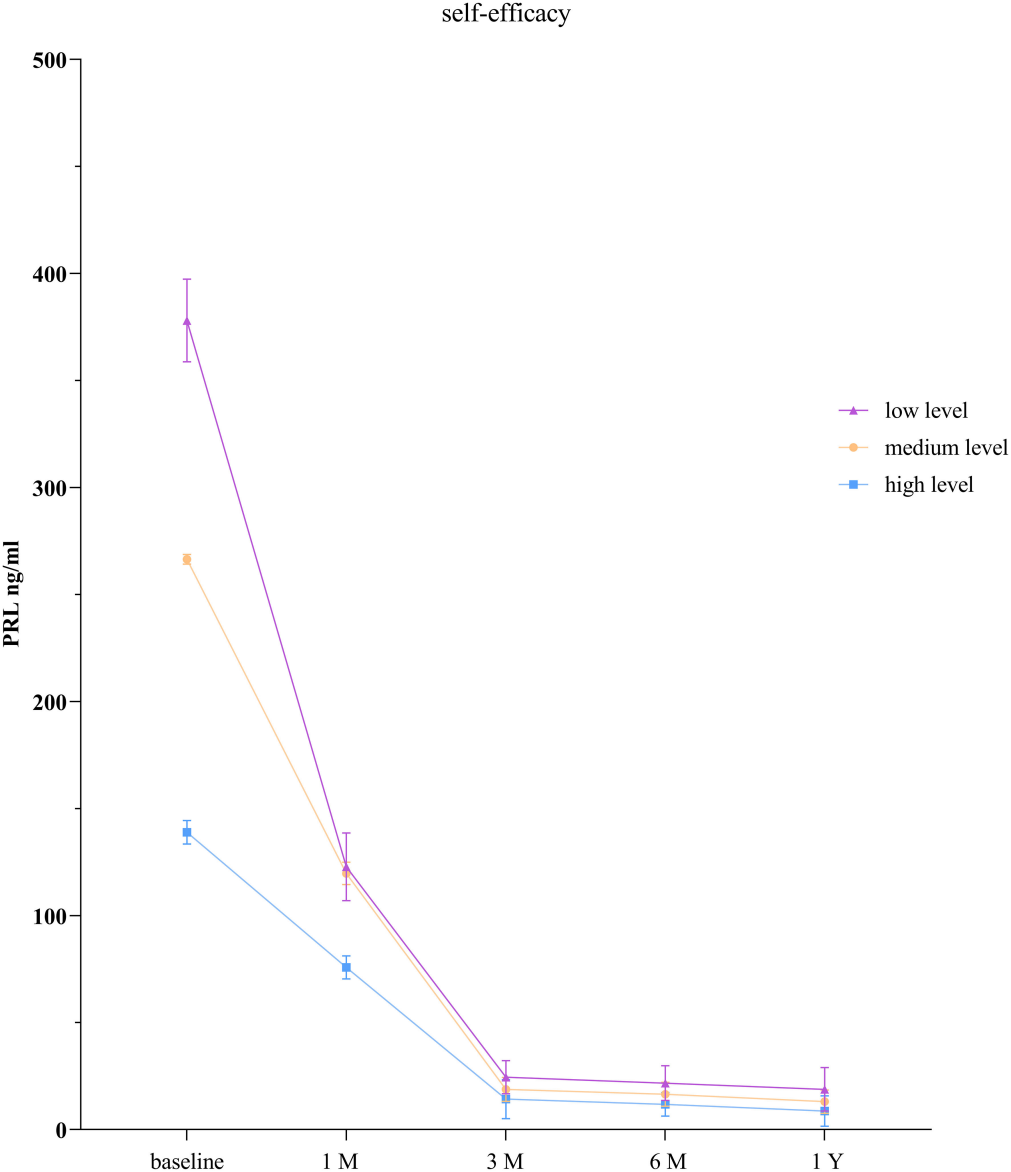
PRL levels in patients with prolactinoma at different levels of self-efficacy.

The GEE model showed that there were statistically significant differences in PRL levels between the low self-efficacy group and the high self-efficacy group as well as between the moderate self-efficacy group and the high self-efficacy group at each time point (*P* < 0.001). There was no statistically significant difference in PRL levels between the low self-efficacy group and the moderate self-efficacy group at the 1-month follow-up (*P* = 0.065), but significant differences were observed at all the other time points (*P* < 0.001; **Table 5**, **Figure 3**).

**Table 5:** Comparison of PRL according to self-efficacy level.

Time	low level (n=96) mean (SD)	medium level (n=49) mean (SD)	high level(n=31) mean (SD)	*P low vs. medium*	*P low vs. high*	*P medium vs. high*
baseline	378.01 (19.28)	266.41 (2.25)	138.90 (5.53)	<0.001	<0.001	<0.001
1 month	122.77 (15.8)	119.70 (5.20)	75.75 (5.39)	0.065	<0.001	<0.001
3 months	24.47 (7.71)	18.78 (5.63)	14.23 (9.10)	<0.001	<0.001	<0.001
6 months	21.70 (8.11)	16.52 (5.56)	11.75 (5.47)	<0.001	<0.001	<0.001
1 year	18.81 (10.21)	13.03 (5.46)	8.63 (7.11)	<0.001	<0.001	<0.001

## 4 Discussion

Prolactinomas account for approximately 50% of all pituitary tumors requiring treatment (24). The 2022 edition of the ICCE/AME consensus states that DAs are the first-line treatment for most prolactinomas (3). These medications can normalize PRL levels in nearly 90% of patients with idiopathic hyperprolactinemia or prolactin microadenomas and in 75-80% of patients with macroprolactinomas (25). Most patients with macroadenomas also experience tumor shrinkage in the early stages of treatment (26), although the exact mechanisms of action are not yet fully understood (27, 28). In this study, we found that PRL levels rapidly decreased in patients with prolactinoma after the 1st month of drug therapy and gradually stabilized at the 3-month, 6-month, and 1-year. PRL levels in patients with prolactinoma may be related to sleep quality, anxiety level, and self-efficacy level. However, due to the limitations of retrospective studies, the causal relationship between psychological factors and prolactin levels could not be clearly established. The rapid decrease in PRL levels is attributed to DAs treatment as prolactinoma cells typically exhibit a high expression of D2-receptor. Long-term DAs therapy leads to significant tumor shrinkage in most case (29).

Our research has shown that prolactin levels in prolactinoma patients are associated with anxiety but not with depression. One possible explanation is that patients with high prolactin levels require continuous administration of DAs to control prolactin levels, and the emotions and behaviors of patients with hyperprolactinemia receiving Das treatment may change (30). Previous study suggested that the occurrence of depression in prolactinoma patients is related to the secondary decrease in estrogen levels caused by hyperprolactinemia (31). However, this was a case series of low quality. Several studies have indicated that patients with hyperprolactinemia are more likely to experience depression, anxiety, and hostility (5, 6). These symptoms are generally alleviated after hyperprolactinemia is corrected, suggesting that prolactin plays a direct role in psychological disorders. Therefore, healthcare professionals should promptly assess the anxiety and depression status of prolactinoma patients receiving long-term DAs treatment and provide psychological counseling. Furthermore, interdisciplinary collaboration is encouraged for the follow-up and symptom management of prolactinoma patients.

Our study revealed that compared to patients without sleep disorders, those with insomnia seemed to have lower PRL levels. A retrospective study reported that 42.3% of hospitalized patients admitted for mental illness and sleep disorders had elevated PRL levels and suggested an association between elevated PRL levels and excessive daytime sleepiness (14), which may be caused by nocturnal insomnia (32). Our study may help explain the relationship between prolactin levels and sleep in prolactinoma patients.

Prolactin can act on different brain regions, including those involved in neural growth; development; protection; sleep; learning; and memory, among others (33). Due to pituitary dysfunction; excessive hormone secretion; and psychological, behavioral, or environmental factors, patients with sellar region tumors may experience various forms of sleep disorders (34). However, due to limitations in the target population and study design, the relationship between prolactin levels in prolactinoma patients and sleep disorders is still controversial. Several studies have indicated that sleep disorders in prolactinoma patients are not directly related to prolactin levels, and they suggest that obesity and higher BMI are associated with sleep disorders in prolactinoma patients (13). Future prospective studies should use objective measures of sleep quality, control for confounding factors, and measure PRL levels at different time points to further explore the relationship between sleep and PRL levels in prolactinoma patients.

Patients with low self-efficacy tend to have higher PRL levels than do those with high self-efficacy. This may be because individuals with high self-efficacy are more likely to develop positive beliefs, increase positive emotions, and actively engage in health-promoting behaviors, leading to better control of PRL levels. Several studies have shown that individuals with higher levels of self-efficacy are more confident in facing adversity or illness and are more likely to adopt a positive attitude toward them (15, 16). Self-efficacy is beneficial for patients to establish a healthy and positive mindset and can improve their confidence in disease treatment (35). Studies have also indicated that an increase in self-efficacy can positively influence the prognosis of patients and improve sleep quality (36). Therefore, interventions aimed at improving sleep self-efficacy should be explored to enhance patients’ sleep quality.

PRL levels in prolactinoma patients are associated with education level. Currently, there is no direct research linking education level to PRL levels, but education level may influence patients’ perception of the disease, choice of treatment plans, and treatment adherence. Prolactinoma patients require the use of DAs to suppress PRL synthesis and secretion, control PRL levels, and prevent tumor recurrence or further growth (3, 37). Patients with higher education levels tend to have better medication compliance, resulting in more effective and stable control of PRL levels.

This study revealed that prolactin (PRL) levels in patients with prolactinoma are correlated with tumor size. Consistent with previous reports (1, 38–41), Osorio et al. (41) demonstrated a significant correlation between preoperative and postoperative tumor volume and PRL levels. For every 1 cubic centimeter increase in preoperative tumor volume, the serum PRL levels increased by 101.31 μg/L. Burke et al. (39) showed a strong correlation between prolactinoma volume and serum PRL levels, while nonfunctioning adenomas did not exhibit a significant correlation. Tumor size has been a subject of debate regarding its relationship with serum PRL levels (42). March et al. (43) reported two patients with galactorrhea and hyperprolactinemia who showed an increase in tumor size without a significant increase in PRL levels. However, the lack of MRI visualization for tumor assessment in these patients may have led to potentially inaccurate detection results. Several researchers have also proposed that the secretory capacity of prolactin is related to the number of intracellular secretory granules, which may not be correlated with tumor volume (44). Therefore, the prolactin levels in some larger prolactinomas may not be high.

Compared to patients who were visiting for the first time, those who had multiple visits for prolactinoma tended to have higher PRL levels. On the one hand, this could be due to tumors developing resistance to DAs, resulting in uncontrolled tumor growth and continued abnormal PRL secretion (45). On the other hand, prolactinomas that have not achieved remission after multiple visits may have a unique molecular composition, leading to higher prolactin levels during tumor growth (41). However, further molecular studies are needed in the future to explore the relationship between the molecular composition of prolactinomas and PRL levels.

This study has several limitations. First, the sample size was relatively small, but the population consisted of patients with prolactinoma and still held a certain representativeness. Second, this was a single-center retrospective study, and future research could include multicenter prospective studies to further investigate the relationships and mechanisms between PRL levels and both prolactinoma and sleep disorders as well as the severity of anxiety. Thirdly, our study lacks a correlation analysis between depression scores and the dosage/exposure of DAs. Due to the constraints of retrospective research and patient medication compliance, it was challenging to accurately collect specific information on the types and doses of DAs taken by the patients. Future prospective studies are needed to analyze the relationship between the dosage/types/duration of DAs medication and depression in prolactinoma patients. Finally, due to limited data, subgroup analysis was not conducted for patients receiving bromocriptine, cabergoline, or combination therapy. Future research should further explore and analyze the impact of DAs on patients’ sleep and psychological well-being.

## 5 Conclusion

In this retrospective study, we analyzed the relationship between prolactin (PRL) levels in patients with prolactinoma and anxiety, self-efficacy, and sleep. Our results showed that PRL levels in prolactinoma patients were related to anxiety, self-efficacy, and sleep but not depression. PRLs were higher in anxious patients than in nonanxious patients. PRL levels were also higher in patients with low self-efficacy than in those with high self-efficacy. Compared with patients without sleep disorders, insomnia patients had higher PRL levels. Future research should further investigate the mechanisms through which psychological and sleep factors affect PRL levels in prolactinoma patients and explore the relationships among psychological factors, sleep quality, and self-efficacy in prolactinoma patients.

## Data availability statement

The raw data supporting the conclusions of this article will be made available by the authors, without undue reservation.

## Ethics statement

Ethical approval was not required for the studies involving humans because this study employs a retrospective research design, where participants are not directly involved physically, and only previously collected data pertaining to them is used for analysis. The studies were conducted in accordance with the local legislation and institutional requirements. Written informed consent for participation was not required from the participants or the participants’ legal guardians/next of kin in accordance with the national legislation and institutional requirements because the study is focused on the utilization of informational data.

## Author contributions

XM: Investigation, Writing – original draft. ZF: Conceptualization, Writing – original draft, Formal analysis. XL: Methodology, Supervision, Writing – review & editing. JW: Methodology, Writing – review & editing. LY: Supervision, Writing – review & editing. SZ: Data curation, Writing – review & editing. YF: Data curation, Writing – review & editing. SH: Conceptualization, Supervision, Writing – review & editing. SX: Conceptualization, Supervision, Writing – review & editing.
